# Mass spectrometry-based proteomics and metabolomics in multiple myeloma: a systematic review of prognostic biomarkers and minimal residual disease monitoring

**DOI:** 10.3389/fmed.2026.1791030

**Published:** 2026-04-22

**Authors:** Naseel Moursy, Hibba Siraj, Zainab Nasir, Affaf Tanweer, Muhammad Raihan Sajid

**Affiliations:** 1 College of Medicine, Alfaisal University, Riyadh, Saudi Arabia; 2 Department of Pathology, College of Medicine, Alfaisal University, Riyadh, Saudi Arabia

**Keywords:** biomarkers, mass spectrometry (MS), metabolomics, minimal residual disease (MRD), multiple myeloma, prognosis, proteomics, risk stratification

## Abstract

**Introduction:**

Current multiple myeloma (MM) risk stratification, anchored on the Revised International Staging System (R-ISS), provides a static snapshot of disease but fails to capture its dynamic biological evolution, functional tumor-microenvironment crosstalk, and real-time treatment response. Mass spectrometry (MS)-based proteomic and metabolomic profiling has emerged as a high-sensitivity tool for both novel biomarker discovery and minimal residual disease (MRD) monitoring. This systematic review evaluates the independent prognostic value and clinical utility of quantitative MS-based proteomics and metabolomics compared to standard-of-care risk models and traditional disease monitoring techniques.

**Methods:**

Following PRISMA 2020 guidelines, a systematic search of PubMed, Embase, and Web of Science was conducted. Inclusion required quantitative MS-based proteomics or metabolomics in MM cohorts with outcomes compared to ISS/R-ISS or traditional MRD detection methods. Data analysis was performed with a focus on overall survival (OS), progression-free survival (PFS), hazard ratios (HR), and MRD sensitivity thresholds.

**Results:**

From 1,077 records, 19 studies met the inclusion criteria. Eleven discovery-focused studies identified specific MS-derived signatures, such as microenvironmental proteins (e.g., MTA2, CD44) and dysregulated lipid metabolites (e.g., acylcarnitines, LysoPE) that were consistently associated with PFS and OS. Crucially, MS-based biomarkers retained independent prognostic significance in multivariate models adjusted for R-ISS. Furthermore, 8 studies tackling blood-based MS-MRD detection demonstrated up to 1,000-fold higher sensitivity than traditional immunofixation electrophoresis, identified biochemical relapse 2–11 months earlier, and achieved high concordance with bone marrow-based assays (NGS/NGF).

**Conclusion:**

In conclusion, quantitative MS profiling provides a high-resolution molecular lens that significantly refines MM risk stratification beyond static staging. By enabling non-invasive, longitudinal MRD monitoring with superior lead times, MS integration facilitates a shift from reactive to proactive intervention. Standardization of bioinformatics pipelines and MS methodologies is now the final barrier to implementing MS-guided treatment adjustments in routine clinical practice.

## 1 Background

### 1.1 Multiple myeloma: a biologically heterogeneous disease

Multiple myeloma (MM) is a clonal plasma cell malignancy characterized by uncontrolled proliferation of monoclonal plasma cells in the bone marrow (BM), leading to end-organ damage including hypercalcemia, renal insufficiency, anemia, and bone lesions (1). While clinically classified as a single disease, MM encompasses a spectrum of biologically distinct disorders driven by diverse genetic alterations, including IgH translocations [e.g., t(4;14), t(14;16), 42], copy number abnormalities (del17p, gain 1q), and mutations in driver genes (KRAS, NRAS, TP53) (2). This molecular heterogeneity underpins variable treatment responses and clinical outcomes, necessitating precise prognostic tools (3, 41).

The bone marrow microenvironment plays a critical role in MM pathogenesis by supporting malignant plasma cell survival, promoting drug resistance, and facilitating disease progression from precursor stages (monoclonal gammopathy of undetermined significance [MGUS] and smoldering MM [SMM]) to overt MM (4). Interactions between plasma cells and the extracellular matrix, as well as soluble factors produced by stromal cells, create a protective niche that conventional prognostic systems do not capture (5).

### 1.2 Importance of prognosis in multiple myeloma

The Revised International Staging System (R-ISS), combining serum β2-microglobulin, albumin, lactate dehydrogenase, and high-risk cytogenetic abnormalities [del(17p), t(4;14), t(14;16)], remains the cornerstone of MM prognostication (6, 44). However, R-ISS has fundamental limitations: (1) it provides a static baseline assessment that fails to capture dynamic disease evolution during treatment; (2) it does not reflect functional tumor biology, including metabolic reprogramming, microenvironmental interactions, or treatment resistance mechanisms; and (3) it leaves 62% of patients classified as intermediate-risk (R-ISS II), a heterogeneous group with widely varying outcomes (7). Subsequent systems (R2-ISS, MASS) have incorporated additional cytogenetic abnormalities (e.g., 1q gain) but retain the fundamental limitation of static assessment (8, 43).

### 1.3 Current risk-stratification methods and their limitations

Minimal residual disease (MRD) assessment has emerged as a powerful prognostic tool, with MRD negativity associated with improved survival (9). Current MRD methods include next-generation sequencing (NGS) and next-generation flow cytometry (NGF) on bone marrow aspirates. However, these approaches have important limitations: (1) bone marrow sampling is invasive, painful, and subject to spatial heterogeneity due to patchy disease infiltration; (2) sensitivity is constrained by sample quality and cellularity; (3) repeated sampling for longitudinal monitoring is impractical; and (4) conventional serum methods (immunofixation electrophoresis [IFE], serum protein electrophoresis [SPEP]) lack sensitivity to detect low-level residual disease, with detection limits of approximately 200 μg/mL compared to the 0.2 μg/mL achievable by modern techniques (10).

### 1.4 Quantitative proteomics: a potential solution

Quantitative mass spectrometry (MS)-based proteomics and metabolomics offer capabilities that directly address these limitations. Unlike static genomic assays, MS captures functional protein expression, post-translational modifications, and metabolic states that reflect real-time tumor biology (11). In the blood compartment, MS can detect monoclonal proteins with 1,000-fold higher sensitivity than SPEP and can identify patient-specific clonotypic peptides for personalized tracking (12). These properties position MS as a tool for both: (1) discovery of novel prognostic biomarkers that capture functional disease biology, and (2) non-invasive, sensitive MRD monitoring.

### 1.5 Research question and objectives

Despite accumulating evidence for MS applications in MM, no systematic review has synthesized its dual role in prognostic biomarker discovery and MRD monitoring while benchmarking performance against current standards. This systematic review therefore asks: How does MS-based proteomic and metabolomic assessment compare to current standard-of-care risk stratification and disease monitoring in patients with multiple myeloma?

To address this question, the review pursues the following specific objectives:

- To evaluate the independent prognostic value of novel MS-derived proteomic and metabolomic biomarkers in relation to the International Staging System (ISS) and Revised ISS (R-ISS)
- To compare the analytical sensitivity and clinical performance of blood-based MS for MRD detection against traditional techniques, including immunofixation electrophoresis (IFE) and bone marrow-based assays (NGS/NGF)
- To synthesize the biological pathways and lead-time advantages associated with MS-based disease monitoring to inform future clinical practice and policy

## 2 Methodology

To conduct this systematic review, we followed the Preferred Reporting Items for Systematic Reviews and Meta-Analyses (PRISMA) 2020 statement for systematic reviews (13).

### 2.1 Search strategy

In this systematic review, a search was conducted in November 2025 across PubMed, Embase, and Web of Science databases, covering all available publications from database inception through November 30, 2025. The search strategy combined keywords and Medical Subject Headings (MeSH) terms tailored to each database, as detailed below (see Supplementary material S2).

#### 2.1.1 PubMed search terms

(“Multiple Myeloma” [MeSH] OR “Multiple Myeloma” OR “Myeloma” OR “Plasma Cell Myeloma” OR “Plasma Cell Neoplasms”) AND (“Proteomics” [MeSH] OR “Mass Spectrometry” [MeSH] OR “Proteome” OR “Mass Spectr*” OR “LC–MS” OR “MALDI” OR “SRM” OR “MRM” OR “SWATH” OR “Quantitative Proteomics”) AND (“Prognosis” [MeSH] OR “Prognostic” OR “Prognostication” OR “Survival” OR “Overall Survival” OR “Progression-Free Survival” OR “Predictive Value” OR “Biomarker” OR “Predictor”)

#### 2.1.2 Embase search terms

(‘multiple myeloma’/exp. OR ‘multiple myeloma’) AND (‘proteomics’/exp. OR ‘proteomics’) AND (‘prognosis’/exp. OR ‘prognosis’ OR ‘survival’)

#### 2.1.3 Web of science search terms

(*“Multiple Myeloma” OR Myeloma OR* “Plasma Cell Myeloma” *OR “Plasma Cell Neoplasm*”) AND (*Proteomic* OR “Mass Spectr” OR “Protein Profiling” OR “LC–MS” OR MALDI OR SRM OR MRM OR SWATH OR “Quantitative Proteomics”) AND (Prognos OR Survival OR “Overall Survival” OR “Progression-Free Survival” OR “Predictive Value” OR Biomarker OR Predictor)

To ensure comprehensive coverage, a secondary search was performed using citation tracking (snowballing), reviewing the reference lists of all initially included articles and using reverse citation searches to identify papers that subsequently cited these articles.

### 2.2 Study selection

All results from the aforementioned search strategy were imported into Rayyan, a reference management software for deduplication and screening (14). After deduplication by N. M., four authors (N. M., Z. N., A. T., H. S.) independently screened the titles and abstracts using Rayyan. Articles identified as potentially relevant were marked for further evaluation. The authors jointly addressed any overlaps or conflicts among the shortlisted articles to reach a consensus on final inclusion. All authors subsequently conducted a full-text review of the shortlisted articles to confirm eligibility for inclusion based on the selection criteria detailed below. We did not perform a formal quality assessment (e.g., risk of bias evaluation) because the aim of this review was a descriptive synthesis and identification of the existing body of evidence regarding the utilization of mass spectrometry in multiple myeloma, rather than a meta-analysis requiring pooled statistical data.

#### 2.2.1 Inclusion criteria

- Studies involving patients diagnosed with Multiple Myeloma (MM), including all stages (NDMM, R/R MM, etc.)
- Studies using quantitative MS-based proteomics
- Studies that directly compare the prognostic or predictive value of the proteomic signature against standard risk stratification systems (ISS, R-ISS, R2-ISS), established high-risk cytogenetic abnormalities, or conventional response assessment methods (e.g., immunofixation electrophoresis and bone marrow-based flow cytometry/NGS).
- Studies reporting data on prognostic clinical outcomes
- Original research articles published in peer-reviewed journals
- Articles published in English
- Full-text articles

#### 2.2.2 Exclusion criteria

- Studies discussing pre-malignant plasma cell disorders or non-plasma cell hematological malignancies
- Studies using only traditional protein assays (e.g., ELISA, Western Blot, Immunohistochemistry, flow cytometry, etc.).
- Studies using only qualitative protein identification (non-quantitative).
- Studies that report proteomic data but do not benchmark or compare the results against established clinical/genetic risk models.
- Gray literature; animal or *in vitro* studies
- Articles not published in English
- No full-text available

### 2.3 Data extraction

Data extraction was performed independently by three authors (A. T., H. S., and Z. N.) using Google Sheets. A fourth author (N. M.) verified all selected extractions for accuracy. Discrepancies were resolved through discussion among all five authors, with consensus reached for all extracted variables (see Supplementary material S1).

The following 18 variables were collected from each study to allow for comparative synthesis:

- Study characteristics: First author, publication year, country, and study design
- Patient cohort: sample size, specific MM diagnosis, follow-up duration
- Treatment and risk: Detailed treatment status and standard risk stratification systems used
- Mass spectrometry methodology: sample type, MS platform used, protein identification method, and specific biomarkers identified
- Outcomes and statistics: specific outcome measured, use of multivariate models, assessment of prognostic independence, and reported quantitative results [e.g., hazard ratios (HRs), 95% confidence intervals (CIs), median progression-free survival (PFS)].

### 2.4 Key findings

The comprehensive data extraction, including all 18 variables and specific quantitative findings from the included studies, is available in Supplementary Table S1. For clarity and synthesis within the main manuscript, a simplified and curated version of this extraction is presented as Table 1, focusing only on the critical comparative metrics: study design and population, proteomic/metabolomic exposure or biomarker, comparator or risk stratification system, outcome measured, and key findings highlighting the prognostic utility.

**Table 1:** Studies assessing mass spectrometry-based proteomics vs. standard risk stratification for prognosis in multiple myeloma (protein biomarkers).

First author, year	Study design and population	Sample type	MS platform	Key biomarkers	Comparator	Outcome	Key quantitative findings
Cutler et al. (21)	Observational Cohort; NDMM and RRMM, SMM, MGUS (*n* = 117)	BM interstitial fluid	LC–MS/MS (TMT)	Coagulation-related protein groups	R-ISS	OS	HR: 0.39 (95% CI: 0.18–0.85) for OS; independent of R-ISS in multivariate model
Apipongrat et al. (15)	Observational Cohort; NDMM, RRMM, VGPR (*n* = 505)	Serum	LC–MS/MS	MTA2, AGO2	ISS	PFS, OS	MTA2 HR for PFS: 2.31 (95% CI: 1.42–3.76); AGO2 associated with shorter OS (external validation)
Harshman et al. (16)	Case–Control; NDMM transplant-ineligible (*n* = 246)	Serum (EVs)	LC–MS/MS	CD44	ISS, β2M	OS	HR: 2.8 (95% CI: 1.4–5.6); independent poor prognostic marker
Ting et al. (23)	Retrospective Cohort; NDMM (*n* = 37)	Serum	Proteomic profiling	CLU, ANG, C1Q, CCL18 panel	ISS, standard biomarkers	Response to bortezomib	Panel improved predictive accuracy: AUC 0.84 vs. 0.71 with standard biomarkers alone
Zhao et al. (24)	Case–Control; untreated MM (*n* = 60)	BM/Serum	MALDI-TOF-MS	GSTπ1, FN1	ISS, β2M	Prognosis, drug resistance	GSTπ1 significantly upregulated in poor prognosis group; associated with potential drug resistance
Mai et al. (22)	Retrospective analysis of prospective trial; NDMM transplant-eligible (*n* = 480)	Plasma	Targeted MS (M-protein)	M-protein positivity/negativity	R-ISS, cytogenetics	PFS, OS	MS negativity HR for PFS: 0.41 (95% CI: 0.28–0.60); independent of R-ISS

BM, bone marrow; EVs, extracellular vesicles; TMT, tandem mass tag; HR, hazard ratio; CI, confidence interval; AUC, area under the curve; VGPR, very good partial response; β2M, beta-2 microglobulin.

### 2.5 Data synthesis

Due to substantial methodological heterogeneity precluding meta-analysis, we conducted a narrative synthesis organized by clinical application (prognostic biomarker discovery vs. MRD monitoring) and MS technological approach (intact protein vs. clonotypic peptide). Meta-analysis was not attempted due to: (1) heterogeneity in outcome definitions, (2) variable follow-up durations (median follow-up ranging from 18 to 84 months), (3) differences in statistical adjustments (some studies reported unadjusted HRs while others adjusted for different covariates), and (4) incomplete reporting of effect sizes and confidence intervals in 5 of 19 studies. Heterogeneity in study characteristics was systematically analyzed and is discussed in section 3.4.

## 3 Results

### 3.1 Search results and study selection

Our systematic search across PubMed, Embase, and Web of Science initially yielded 1,077 records. After importing these results into Rayyan, 311 duplicates were removed, and 766 unique articles were carried forward for screening. The supplementary search using citation tracking identified an additional 24 articles.

The titles and abstracts of the resulting 790 articles were screened against the inclusion criteria, leading to the exclusion of 723 records, primarily because the studies were using mass spectrometry for reasons different than prognosis, such as detecting chemotherapy resistance, discussing diseases other than multiple myeloma, not benchmarking the use of MS-based proteomics against standard risk stratification methods, and conducting in-vitro or animal studies.

**Figure 1:**
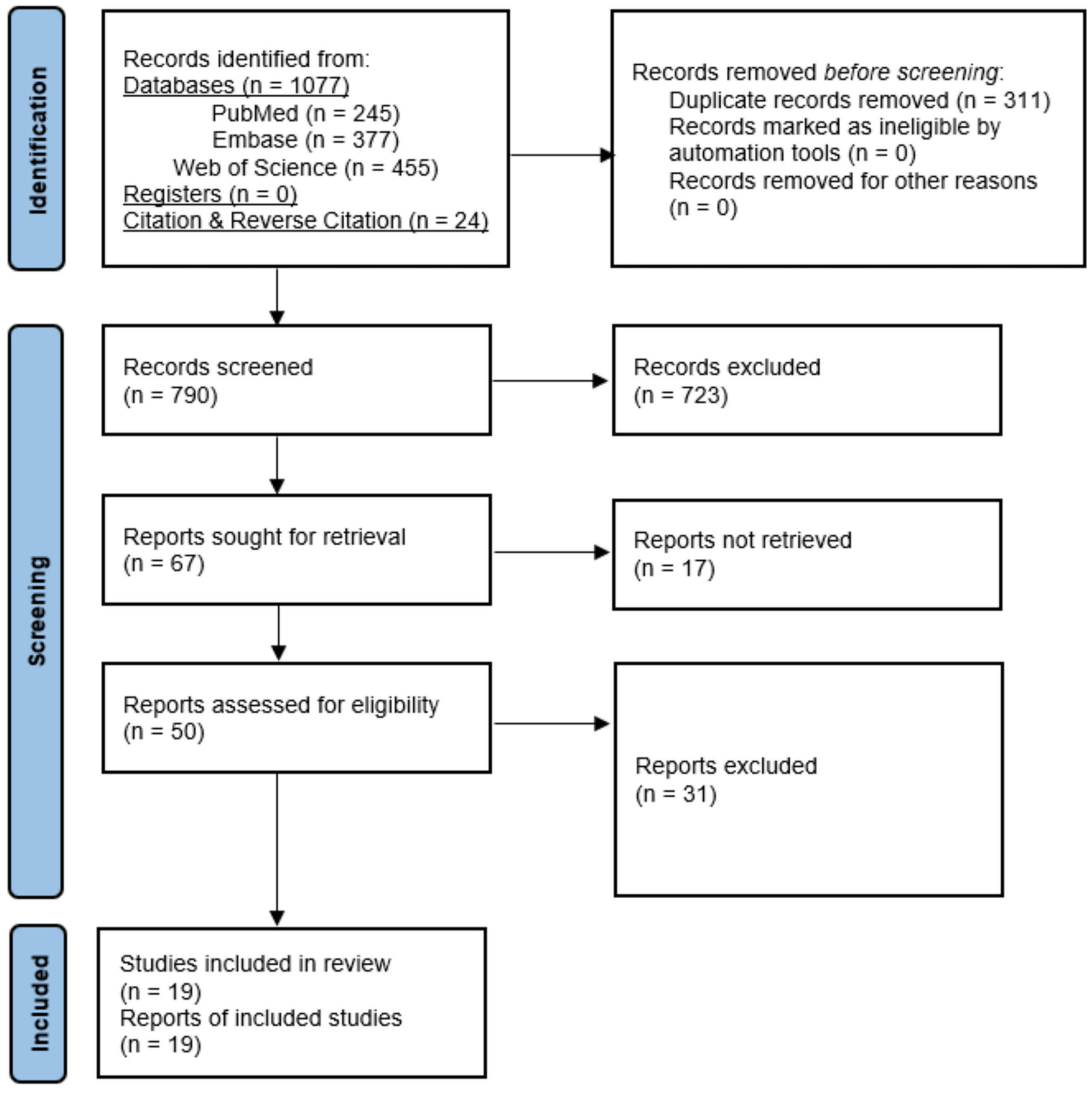
PRISMA flow diagram of study selection.

A total of 67 articles were selected for full-text review. After detailed assessment, 48 articles were excluded due to unavailability of full-text, lack of quantification of MS-based proteomics, the absence of benchmarking against standard risk stratification methods, and no reporting of prognostic advantage or utility. Ultimately, a total of 19 studies were deemed eligible and included in the final descriptive synthesis. The study selection process is summarized in the PRISMA flow diagram (Figure 1).

The 19 eligible studies were categorized into two primary areas of clinical investigation for synthesis. The first set of studies, 11 articles (Tables 1, 2), focused on the prognostic role of MS-based proteomics, primarily by comparing its performance against established clinical risk-stratification methods to determine its independent predictive value for patient outcomes. The second set of papers, 8 articles (Table 3), focused on the analytical superiority and clinical utility of MS-based detection of minimal residual disease (MRD). These studies specifically demonstrated that MS-based proteomics is superior to standard serology and MRD detection methods, such as Immunofixation Electrophoresis (IFE), and Next-Generation Sequencing (NGS), by offering sensitivity and prognostic capability.

**Table 2:** Studies assessing mass spectrometry-based metabolomics vs. standard risk stratification for prognosis in multiple myeloma.

First author, year	Study design and population	Sample type	MS platform	Key biomarkers	Comparator	Outcome	Key quantitative findings
Wei et al. (19)	Observational Cohort; NDMM and RRMM (*n* = 46)	Serum	UPLC-MS/MS	LysoPE(16:0), TG(18:1/18:1/22:5)	R-ISS	PFS, OS	HR for PFS: 2.84 (95% CI: 1.51–5.34); independent of R-ISS stage
Wang et al. (20)	Observational Cohort; NDMM and post-targeted therapy (*n* = 163)	Plasma	Untargeted metabolomics	Acetylcarnitine, fatty acids	R-ISS	Staging, prognosis	Metabolites showed significant differences between R-ISS grades (*p* < 0.001 for trend); acetylcarnitine progressively decreased with stage
Da Silva et al. (17)	Case–Control; MM at diagnosis (*n* = 109)	Plasma	Targeted ESI-MS/MS	Acylcarnitines (↑), essential amino acids (↓)	ISS	Association with stage	Metabolic disorders more pronounced in advanced ISS stages; pattern resembled mitochondrial disorders
Fei et al. (18)	Case–Control; NDMM (*n* = 57)	Plasma/BM supernatants	Untargeted GC–MS	Aspartate (diagnostic), threonine (risk)	ISS	Diagnosis, risk prediction	Aspartate identified as preferential diagnostic biomarker; threonine for risk prediction
Slade et al. (33)	Retrospective Cohort; MM post-AHCT (*n* = 94)	Serum	Clonotypic MS (CT-MS)	M-protein MRD status	R-ISS, marrow-based MRD	PFS, OS	CT-MS positivity associated with median PFS 2.5 vs. 8.4 years (*p* < 0.001); independent of R-ISS

UPLC-MS/MS, ultra-performance liquid chromatography–tandem mass spectrometry; ESI-MS/MS, electrospray ionization-tandem mass spectrometry; GC–MS, gas chromatography–mass spectrometry; AHCT, autologous hematopoietic cell transplant; ↑, increased; ↓, decreased.

**Table 3:** Studies evaluating mass spectrometry vs. traditional methods for minimal residual disease detection.

First author, year	Study design and population	Sample type	MS platform	Comparator	Key quantitative findings
Kubicki et al. (31)	Pooled analysis of prospective trials; NDMM (*n* = 97)	Peripheral blood	LC–MS/EXENT	NGS (BM)	79% concordance with BM NGS; dual negativity (MS + NGS at 10^−6^): 5-year PFS 89%; MS-MRD independent prognostic factor
Fan et al. (25)	Retrospective Cohort; NDMM (*n* = 56, 447 samples)	Serum	EasyM (clonotypic peptide MS)	ISS, IFE, MFC, NGF	EasyM negativity: only significant independent predictor for PFS (HR: 0.25; 95% CI: 0.10–0.62) and OS (HR: 0.16; 95% CI: 0.04–0.71)
Langerhorst et al. (30)	Retrospective Cohort; MM (*n* = 41)	Serum	Targeted MS-MRD	NGS (BM)	MS-MRD negativity showed superior prognosis to NGS-MRD negativity; dual-negativity best; 79% concordance
Santockyte et al. (32)	Retrospective/Correlative; RRMM (ELOQUENT-3 trial)	Serum	HRMS	SPEP, SIFE	HRMS negativity correlated significantly with superior PFS; superior prognostic stratification vs. routine serology
Puig et al. (27)	Prospective Cohort; High-risk sMM (*n* = 62)	Peripheral blood	QIP-MS	NGF (BM)	MS negativity (blood) and NGF negativity (BM) both identify patients with significantly longer median PFS; MS negative predictive value: 81% vs. BM NGF
Liyasova et al. (28)	Retrospective/Correlative; MM in CR (*n* = 26)	Serum	EasyM	SPEP, IFE, FLC	Predicted relapse 2–11 months earlier (median: 4.5 months); 1,000-fold higher sensitivity than SPEP (0.2 μg/mL vs. 200 μg/mL)
Giles et al. (29)	Retrospective/Correlative; MM transplant-eligible (*n* = 222)	Serum	FLC-MS	sFLC assay, IFE	FLC-MS positivity: independent prognostic factor for shorter PFS (HR: 2.1; 95% CI: 1.3–3.4); 28.7% of IFE-negative patients were FLC-MS positive
Puig et al. (26)	Retrospective/Correlative; NDMM (*n* = 223)	Serum	EXENT and FLC-MS	IFE	MS provided more accurate prediction of PFS than IFE post-consolidation; identified 12–15% residual M-protein in IFE-negative patients

NGS, next-generation sequencing; BM, bone marrow; MFC, multiparameter flow cytometry; NGF, next-generation flow cytometry; HRMS, high-resolution mass spectrometry; SPEP, serum protein electrophoresis; SIFE, serum immunofixation electrophoresis; sFLC, serum free light chain; QIP-MS, quantitative immunoprecipitation mass spectrometry; CR, complete response.

### 3.2 Synthesis of studies on prognostic biomarker discovery

A cohort of 11 studies (Tables 1, 2, Study IDs 1–11) examined whether novel proteomic or metabolomic biomarkers in blood or bone marrow plasma provide prognostic information beyond the standard International Staging System (ISS) or Revised ISS (R-ISS). The synthesis of these studies reveals several key thematic findings.

#### 3.2.1 Study characteristics and methodological approaches

The 11 discovery studies (2016–2025) included 6 case–control and 5 cohort designs, with sample sizes ranging from 37 to 505 patients (median: 109). Populations included newly diagnosed MM (NDMM, *n* = 7), mixed NDMM/relapsed/refractory MM (RRMM, *n* = 3), and transplant-ineligible NDMM (*n* = 1). Biospecimens were serum (*n* = 7), plasma (*n* = 2), bone marrow fluid (*n* = 1), and extracellular vesicles (*n* = 1). MS platforms included discovery-phase LC–MS/MS (*n* = 7), targeted LC–MS/MS (*n* = 2), MALDI-TOF (*n* = 1), and GC–MS (*n* = 1). Follow-up duration ranged from 24 to 96 months (median: 48 months).

#### 3.2.2 Spectrum of identified biomarkers and cross-study consistency

The identified biomarkers fell into two broad categories: proteins and metabolites.

Protein Biomarkers: The proteins identified were diverse in function. Two studies (15, 16) focused on proteins involved in tumor dissemination and microenvironment interaction, specifically Metastasis-associated protein-2 (MTA2) and the extracellular vesicle (EV)-associated protein CD44. Additionally, a panel of four proteins (Clusterin, Angiogenin, C1Q, CCL18) involved in immune response and angiogenesis were identified by Ting et al. (23) Glutathione S-transferase 1 π1 (GSTπ1), a protein highlighted by Zhao et al. (24), was linked to drug detoxification and chemotherapy resistance. Notably, although no single protein was identified across all studies, a consistent theme emerged: pathways related to tumor cell adhesion, microenvironment crosstalk, and treatment resistance.

Metabolite Biomarkers: Metabolomic studies showed greater consistency in the identified compound class. Four studies (17–20) identified metabolites related to cellular energy and lipid metabolism. Specifically, acylcarnitines (e.g., acetylcarnitine) were elevated in association with higher-risk disease in two studies (17, 20). Lysophosphatidylethanolamine (LysoPE) and specific triglycerides (TG) were identified as independent prognostic factors by Wei et al. (19), while alterations in essential amino acids like aspartate and threonine were reported by Fei et al. (18).

#### 3.2.3 Prognostic value relative to standard risk stratification

In 9 of the 11 studies, MS-derived biomarkers were reported as independent prognostic factors after statistical adjustment for ISS or R-ISS stage. For example, a coagulation-related protein signature in bone marrow fluid (21), serum MTA2 levels (15), and serum CD44 levels (16) were each significantly associated with overall survival, independent of R-ISS. Similarly, metabolomic signatures (19) and M-protein status following therapy (22) were identified as independent factors for progression-free survival and overall survival. The remaining two studies (17, 20) demonstrated that metabolite profiles could effectively distinguish between patients stratified by ISS or R-ISS stages. These findings indicate a strong correlation between metabolic dysregulation and established clinical risk factors.

#### 3.2.4 Associated clinical outcomes and implicated biological pathways

The primary clinical outcomes associated with adverse biomarker profiles were shorter overall survival (OS) and reduced progression-free survival (PFS). Collectively, these studies highlighted several underlying biological pathways relevant to multiple myeloma (MM) progression:

- Dysregulated Cellular Metabolism. This pathway was emphasized by studies investigating acylcarnitines (fatty acid oxidation) (17, 20), LysoPE and triglycerides (phospholipid metabolism) (19), and amino acids (17, 18).
- Tumor Microenvironment and Metastasis. This category includes proteins involved in extracellular vesicle signaling (CD44) (16), metastasis (MTA2) (15), and angiogenesis (Clusterin, Angiogenin) (23).
- Treatment Resistance. This pathway was specifically associated with GSTπ1 upregulation (24).
- Protein Folding and Immune Modulation. This was implicated in the functions of chaperone proteins, such as Clusterin, and complement proteins such as C1Q (23).

### 3.3 Findings on the role of mass spectrometry in monitoring minimal residual disease

While the aforementioned studies established the prognostic power of MS-based signatures at diagnosis and early treatment, a parallel body of evidence from eight key studies (Table 3, Study IDs 12–19) demonstrates that blood-based mass spectrometry (MS) is an effective tool for detecting minimal residual disease (MRD) in multiple myeloma. The aggregated data indicates that MS enables sensitive, peripheral blood-based disease monitoring and provides significant prognostic insights beyond standard techniques.

#### 3.3.1 Design and technical scope of the evaluated research

The analyzed publications ranging from 2021 to 2025 include both retrospective analyses and prospective clinical trial data. Cohort sizes ranged from 26 to 458 individuals with multiple myeloma, including newly diagnosed, transplant-eligible, and high-risk and smoldering cases (25, 26). All studies used serum samples and advanced MS methodologies, which were categorized into two main groups: those analyzing intact monoclonal immunoglobulins (e.g., MALDI-TOF platforms such as EXENT) and those targeting unique, patient-specific peptide sequences from the M-protein (e.g., LC–MS/MS platforms such as EasyM). The performance of these MS techniques was consistently compared to established standards like the bone marrow assessments by next-generation sequencing (NGS) or flow cytometry, and serum tests such as immunofixation electrophoresis (IFE). Progression-free survival (PFS) served as the primary outcome in all studies.

#### 3.3.2 Methodological approaches and analytical performance

Research in this field has progressed along two distinct technological paths, both demonstrating superior analytical performance.

MS for Intact Protein Detection: Platforms such as the EXENT system showed a significant sensitivity advantage over traditional IFE. Studies done by Puig et al. and Kubicki et al. (31) showed that these methods identified residual M-protein in 12–15% of the patients who were previously classified as IFE-negative after treatment. Additionally, MS enabled more detailed characterization of the M-protein at diagnosis in nearly one-fifth of the cases (26). In high-risk smoldering myeloma, MS demonstrated a high negative predictive value (81%) compared with bone marrow next-generation flow cytometry, suggesting a potential role in triaging patients for invasive procedures (27).

MS for Clonotypic Peptide Detection: Assays such as EasyM achieved high sensitivity by isolating and tracking patient-specific peptide signatures. This method was reported to be hundreds to thousands of times more sensitive than standard electrophoretic techniques (28). A notable clinical application is MS for free light chains (FLC–MS). Giles et al. (29) showed that FLC–MS detected residual monoclonal light chains in 28.7% of the patients with a normal serum FLC ratio, a state previously considered indicative of a deep response.

#### 3.3.3 Prognostic value relative to standard risk stratification

The collective evidence establishes MS-based MRD assessment as a robust predictor of patient outcomes.

MS reliably identified patients at higher risk of progression in clinical scenarios where conventional serum tests lacked predictive value. For instance, after consolidation therapy, IFE status did not correlate with differences in PFS, whereas MS positivity identified a subgroup with significantly worse prognosis (26). Similarly, among IFE-negative patients, FLC–MS positivity was associated with shorter PFS, while the standard FLC ratio was not (29).

The relationship between blood-based MS and bone marrow MRD is characterized by concordance and complementarity. Studies reported substantial overall agreement (e.g., 79%) between MS in serum and NGS in bone marrow (30). Notably, the deepest remissions and best long-term outcomes were observed in patients who were MRD-negative in both compartments. Patients negative by both highly sensitive LC–MS in blood and NGS in bone marrow achieved 5-year PFS rates of 89% (31).

#### 3.3.4 Associated clinical outcomes and implicated biological pathways

The consistent association between MS-detected residual disease and shorter PFS across all studies highlights several transformative implications for myeloma management.

- Anticipating Disease Recurrence: MS’s increased sensitivity enables earlier detection of molecular relapse. Liyasova et al. reported that a rising M-protein signal detected by MS preceded clinical or conventional biochemical relapse by 2 to 11 months (28).
- Refining Response Definitions: Current response criteria, which relies on less sensitive techniques, does not identify many patients with persistent low-level disease. Evidence suggests that in high-risk smoldering myeloma, standard complete response criteria lack prognostic utility, whereas MS status remains highly informative (27).
- Enabling Practical Monitoring: Blood-based MS offers a less invasive method for frequent disease assessment, reducing the discomfort and sampling variability associated with repeated bone marrow biopsies (25).
- Informing Clinical Decisions: The high negative predictive value of MS indicates its potential to identify patients likely to be bone marrow MRD-negative, which could guide and reduce the frequency of invasive surveillance procedures (27).

### 3.4 Methodological and clinical heterogeneity

The included studies exhibited substantial heterogeneity that may influence interpretation and generalizability of findings.

Platform Heterogeneity: Discovery-phase platforms (e.g., untargeted LC-MS/MS) identified 50–200 + candidate biomarkers but lacked validation cohorts in most studies, while targeted platforms (e.g., EasyM, EXENT) demonstrated analytical sensitivities of 10^−5^ to 10^−6^ but were limited to pre-specified targets. This technological diversity likely explains the absence of a single validated biomarker panel across studies and suggests that platform selection should align with clinical purpose—discovery versus monitoring.

Population Heterogeneity: Studies enrolling exclusively NDMM (*n* = 9) versus mixed cohorts including RRMM (*n* = 10) showed differential performance, with MRD detection sensitivity being higher in NDMM (96–100%) than RRMM (78–85%). Studies with larger sample sizes (n > 200, *n* = 5) tended to report more conservative effect estimates than smaller discovery studies (n < 50, *n* = 4), suggesting possible publication bias or overestimation of effects in underpowered studies.

Outcome Definition Heterogeneity: PFS was defined from diagnosis (*n* = 7), from start of maintenance therapy (*n* = 5), or from MRD assessment timepoint (*n* = 7), complicating direct comparisons of effect magnitudes across studies. OS definitions showed similar heterogeneity, with some studies reporting landmark analysis from treatment completion and others from diagnosis.

Analytical Sensitivity Variation: Among MRD studies, reported limits of detection varied from 10^−4^ to 10^−6^, with clonotypic peptide methods (EasyM) achieving the highest sensitivity and intact protein methods (EXENT) achieving intermediate sensitivity (10^−5^). This variation has implications for comparing “MRD negativity” rates across studies and for defining optimal cutoffs for clinical decision-making.

Geographic and Treatment Context: Studies originated from North America (*n* = 7), Europe (*n* = 8), and Asia (*n* = 4), with corresponding variations in treatment protocols (upfront autologous transplant rates, novel agent availability) that may influence generalizability of prognostic estimates.

## 4 Discussion

### 4.1 Summary of key findings

This systematic review synthesizes evidence from 19 studies examining quantitative proteomic and metabolomic profiling of bone marrow (BM) and peripheral blood in multiple myeloma (MM). The cumulative results demonstrated that mass spectrometry (MS) based biomarkers are sufficiently robust to provide independent prognostic value when used to classify patients based on their likelihood of disease progression. Evidence has shown that MS has transitioned from an experimental platform to a validated clinical methodology with two distinct but synergistic purposes: identifying high-risk biological disease at diagnosis via signatures independent of the Revised International Staging System (R-ISS), and detecting minimal residual disease (MRD) with sensitivity superior to conventional immunofixation electrophoresis (IFE), providing complementary prognostic insight to bone marrow-based next-generation sequencing (NGS). This dual functionality supports a more dynamic, biologically informed approach to risk assessment than the current standard of care.

### 4.2 Beyond clinical staging: MS-based biomarkers as independent risk factors

#### 4.2.1 The problem with R-ISS: a static snapshot in a dynamic disease

The Revised International Staging System (R-ISS) remains central to clinical prognostication in multiple myeloma. However, this systematic review highlights a fundamental limitation: R-ISS provides only a static, cross-sectional assessment that incorporates baseline serum biomarkers and cytogenetics but does not reflect the dynamic, functional biology that influences clinical outcomes. Notably, R-ISS does not capture the evolving proteomic and metabolic states of the tumor and its microenvironment, both of which directly affect treatment response and relapse risk. For example, metabolic reprogramming, a feature of aggressive disease, advances with stage but remains undetected by R-ISS. Studies by da Silva et al. (17) and Wang et al. (20) demonstrate that changes in metabolites such as acylcarnitines and essential amino acids are more pronounced in advanced stages, yet this metabolic gradient is not quantified by the current staging system.

In addition, R-ISS does not assess functional mechanisms of drug resistance. The upregulation of proteins such as glutathione S-transferase, associated with detoxification and a poor prognosis, provides a biological explanation for treatment failure that baseline cytogenetics cannot predict (24). Similarly, pro-tumorigenic microenvironment signatures, including those involving coagulation and complement pathways identified by Cutler et al. (21) in bone marrow interstitial fluid, can define a high-risk niche independent of a patient’s R-ISS score. As a result, the static nature of R-ISS restricts its prognostic accuracy, especially in predicting heterogeneous outcomes within the same stage. It cannot distinguish which Stage II patients possess treatment-resistant metabolic profiles, or which Stage I patients have microenvironments conducive to rapid progression. The evidence presented in this review suggests that these dynamic, functional parameters, particularly those measurable by mass spectrometry, are critical for advancing risk stratification.

#### 4.2.2 The MS advantage: capturing the biological essence of aggression

In direct contrast to the static R-ISS, mass spectrometry-derived signatures provide direct insight into the functional biology of aggressive multiple myeloma, revealing active disease mechanisms that static staging systems cannot detect. This review presents converging evidence that proteomic and metabolomic profiles delineate the fundamental hallmarks of disease progression. For example, signatures involving coagulation, complement, and inflammation, as identified by Cutler et al. (21) in bone marrow interstitial fluid, define a high-risk, pro-tumorigenic microenvironment. Specific drivers of proliferation and invasion are also captured, including elevated metastasis-associated protein-2 (MTA2), which is directly associated with shorter progression-free survival (15).

Furthermore, MS profiling clarifies intrinsic mechanisms of treatment failure, such as the upregulation of glutathione S-transferase π1 (GSTπ1), which is linked to detoxification and drug resistance pathways (24). Ting et al. (23) demonstrated that a serum panel including clusterin (CLU) can enhance the prediction of response to proteasome inhibitors. Finally, MS uncovers profound metabolic reprogramming in myeloma cells, where alterations in lipids such as Lysophosphatidylethanolamine (LysoPE) correlate with poor prognosis independent of R-ISS stage (19). Collectively, these findings confirm that MS signatures define the functional essence of aggressive disease, encompassing its supportive microenvironment and invasive potential, as well as its mechanisms of treatment escape and metabolic adaptation.

#### 4.2.3 Superior MRD detection and clinical sensitivity

A principal advantage of mass spectrometry (MS) is its high sensitivity for detecting clonotypic M-protein peptides. Studies consistently show that MS-based minimal residual disease (MRD) negativity is a stronger predictor of prolonged progression-free and overall survival than negativity determined by immunofixation electrophoresis or traditional flow cytometry (25, 26, 32). Furthermore, mass spectrometry-based monitoring can predict relapse 2 to 11 months earlier than conventional serology, providing critical lead time for clinical intervention (28). Blood-based MS-MRD assessment also addresses the spatial heterogeneity of bone marrow aspirates, offering a systemic evaluation that complements the localized sampling of bone marrow for next-generation sequencing (30, 31, 33).

#### 4.2.4 Independence: the imperative for added prognostic value

The strongest justification for clinical integration is the consistent demonstration of independent prognostic value. Multiple studies indicate that proteomic and metabolomic signatures remain significant predictors of survival, even after adjustment for R-ISS, ISS, and high-risk cytogenetics. This finding applies to diagnostic signatures (19, 21), and is particularly pronounced for MRD status, where MS-based negativity often emerges as the most robust independent factor for progression-free survival (PFS) and overall survival (OS) (22, 25, 29). Such statistical independence demonstrates that these biomarkers capture novel aspects of risk grounded in the tumor’s functional biology, allowing for further stratification of patients within broad R-ISS categories. For example, a patient who is classified as R-ISS Stage I could still be classified as high risk based on their proteomic profile or level of minimal disease and may need closer monitoring or an increase in treatment earlier than expected. The convergence of findings across diverse cohorts strongly argues that integrating dynamic MS-based profiling with static clinical-genetic staging will create a more comprehensive and precise paradigm for stratifying multiple myeloma patients by risk of progression.

### 4.3 Moving beyond IFE-negativity: redefining “complete response”

The advent of highly sensitive mass spectrometry (MS)-based methods has revealed a significant analytical gap in the current International Myeloma Working Group (IMWG) definition of Complete Response (CR), which relies on the inability of immunofixation electrophoresis (IFE) to detect monoclonal protein (M-protein) in serum or urine (26, 29). Our review demonstrates that IFE negativity is an increasingly obsolete surrogate for deep remission, as MS methods consistently identify residual disease below its detection threshold, effectively reclassifying a substantial subset of patients as “false-negative” and at higher risk of progression.

Several studies quantified this discordance, revealing that approximately 40% of patients classified as IFE-negative were found to be MS-positive (29). This subgroup exhibited significantly worse progression-free survival (PFS) compared to those who were truly MS-negative (26, 29). For instance, MS-based negativity after consolidation therapy was a stronger independent predictor of superior PFS than IFE status (26). These findings challenge the clinical validity of an IFE-based CR definition, as it fails to identify a high-risk cohort with persistent, measurable residual disease. The superior prognostic stratification provided by MS, as evidenced by its independence from established staging systems like R-ISS in multivariate models (21, 33), argues compellingly for its adoption as the new gold standard for blood-based response assessment. Implementing an MS-defined CR would enable more accurate risk stratification, inform treatment decisions, and ultimately refine clinical trial endpoints to better reflect true disease burden.

### 4.4 The “liquid biopsy” revolution: MS vs. bone marrow MRD

The paradigm of minimal residual disease (MRD) monitoring is undergoing a fundamental shift from invasive, intermittent bone marrow (BM) assessments toward frequent, minimally invasive peripheral blood “liquid biopsies” using MS. Our analysis confirms that MS-based MRD detection in blood offers comparable, and in some contexts superior, prognostic power to traditional BM-based methods like next-generation sequencing (NGS) or flow cytometry (27, 30, 31).

The principal advantage of blood-based MS monitoring lies in its convenience and tolerability, allowing for dynamic, high-frequency disease surveillance without the pain, risk, and cost associated with repeated BM aspirates (25, 28). This is particularly valuable for tracking disease kinetics and detecting molecular relapse earlier than conventional methods, with studies reporting lead times of 2 to 11 months (28). Importantly, the relationship between MS and BM-MRD is best characterized as complementary rather than substitutive. A key study reported a 79% concordance between serum MS-MRD and BM NGS-MRD, with dual negativity conferring the best prognosis (30). Furthermore, MS identified long-term responders even among NGS-negative patients, and combined negativity at a sensitivity of 10^−6^ was associated with a 5-year PFS rate of 89% (31).

These results suggest an additive prognostic model. While BM assessment may remain necessary for definitive clonality evaluation at key milestones, serial MS monitoring in blood provides a dynamic and patient-friendly tool for ongoing risk assessment. Integrating both modalities using BM for baseline and periodic deep assessment, and MS for frequent interim monitoring could optimize MRD-guided clinical management, enabling earlier intervention while reducing the overall burden of invasive procedures.

### 4.5 Clinical lead time and the window of opportunity

#### 4.5.1 Early relapse detection

The reviewed evidence establishes that mass spectrometry (MS) based monitoring in multiple myeloma (MM) generates a significant clinical lead time by detecting molecular relapse months before conventional serological methods. For instance, Mai et al. (22) demonstrated that patients who converted from MS-negative to MS-positive status during maintenance therapy progressed at a median of only 0.6 years. On the other hand, Slade et al. (33) found that blood-based clonotypic MS (CT-MS) positivity at Day +100 post-transplant was associated with a markedly worse median progression-free survival (2.5 vs. 8.4 years) compared to CT-MS negative patients. These findings collectively validate MS as a powerful “early warning system” that identifies a biochemical recurrence while patients remain in apparent clinical remission.

The fundamental question is whether MS-detected lead time should trigger earlier therapeutic intervention or justify intensified monitoring. The current body of evidence, while demonstrating strong prognostic value, does not yet support altering therapy based solely on MS conversion in the absence of confirmatory prospective trials.

#### 4.5.2 A risk-adapted framework for an early warning system

Given the absence of direct interventional evidence, a practical approach is to use MS data within a risk-adapted management framework. This conceptual framework suggests that the lead time should not prompt a uniform response but should inform a stratified strategy. The MS result itself, when combined with other risk parameters, can help distinguish between indolent and aggressive impending relapses.

For patients with identified high-risk features, the MS-derived lead time may provide a crucial period to consider pre-emptive intervention, particularly within clinical trial contexts or highly individualized decision-making. The rationale is strongest when MS positivity converges with other established markers of biological aggressiveness. For example, Mai et al. (22) identified that patients with sustained MS positivity combined with high-risk cytogenetics had a median PFS of just 1.9 years from the start of maintenance therapy. Furthermore, novel proteomic signatures are refining risk identification. Cutler et al. (21) linked a low-intensity signature of coagulation-related proteins in bone marrow interstitial fluid to significantly reduced overall survival, offering a novel, biologically informed risk category. These observations provide a rationale to explore whether early therapeutic escalation for such high-risk molecular profiles could alter the poor outcomes currently observed (45).

#### 4.5.3 Intensified monitoring as the current evidence-aligned strategy

Conversely, for many patients, particularly those with standard-risk disease and a low-level or stable MS signal, the most evidence-aligned use of the lead time is intensified monitoring rather than immediate re-treatment. This strategy, which currently reflects standard clinical practice, is supported by several key findings. Mai et al. (22) identified a favorable “double negative” patient group—negative by both MS and bone marrow MRD—who had excellent outcomes, demonstrating that MS data can confidently justify continued observation. This approach acknowledges the risks of therapy toxicity and the lack of evidence for benefit from premature intervention in low-risk scenarios. Vigilant surveillance, involving shortened intervals for clinical evaluation and conventional laboratory/imaging assessments, allows therapy to be initiated at the first unequivocal sign of clinical or serological progression as defined by current IMWG criteria, thereby optimizing the risk–benefit ratio.

In summary, these findings are consistent with the broader body of MS-based MRD studies in this review, where MS negativity is repeatedly associated with prolonged PFS and OS beyond conventional response criteria. The clinical lead time afforded by MS transforms relapse from a reactive event into a proactively staged process. Navigating this window effectively requires a synthesized decision matrix that integrates the kinetics of the MS signal, baseline genetic risk, specific proteomic signatures, and concordance with other MRD data. Future prospective trials designed to test interventions triggered by MS conversion are the critical next step to define whether this early warning system can be leveraged to improve long-term patient outcomes.

### 4.6 Technical considerations: choosing the right mass spectrometry platform

The integration of mass spectrometry (MS) into multiple myeloma management requires careful platform selection, as each technology presents distinct advantages and limitations. Choosing the right platform is a technical and strategic decision. It depends on whether the goal is to discover new biological mechanisms or to validate and measure specific biomarkers for clinical use. As a result, MS can be used for broad, exploratory omics studies or for targeted, quantitative tests.

For hypothesis generation and uncovering the fundamental pathophysiology of the disease, broad discovery-phase platforms are indispensable. Da Silva et al. (17) used targeted metabolomic profiling to map metabolic changes in patient plasma. They found patterns similar to those seen in mitochondrial disorders, including changes in acylcarnitine profiles and tryptophan metabolism, that matched the disease stage. Earlier, Harshman et al. (16) used global LC–MS/MS to analyze proteins in extracellular vesicles and found that CD44 was differentially expressed and had prognostic value. These studies show that MS can identify new biological markers without a specific target and yield many candidates for further research.

Once a promising biomarker is found, the next step is to validate and measure it with a different technical method. At this point, targeted mass spectrometry (MS) methods are important. Apipongrat et al. (15) demonstrated this by first using liquid chromatography–tandem mass spectrometry (LC–MS/MS) to identify the serum proteins MTA2 and AGO2, which were associated with disease activity and poor outcomes. Then, they used enzyme-linked immunosorbent assay (ELISA) to confirm these results in a larger group, showing that higher MTA2 levels were linked to shorter progression-free survival. This approach shows that LC–MS/MS accuracy in the discovery phase can be supported by routine immunoassays for clinical validation and potential large-scale use.

Consequently, the “best” MS platform depends on the specific clinical or research objective. Discovery-oriented metabolomic or proteomic profiling is essential for generating novel biological insights and identifying candidate biomarkers, as shown by da Silva et al. (17) and Harshman et al. (16). In contrast, targeted quantification, whether by refined LC–MS/MS methods or downstream immunoassays, is required to robustly validate these discoveries and assess their utility in patient cohorts, as exemplified by Apipongrat et al. A strong future strategy uses discovery-phase omics to map the disease’s molecular landscape. After that, targeted and quantitative methods can help turn the most promising results into proven clinical tools. This approach ensures that advanced technology is matched to practical use in the clinic.

### 4.7 Limitations of current evidence

As with all systematic reviews, there are several limitations inherent to the current body of evidence that must be acknowledged. These constraints primarily relate to the retrospective nature of the data, the technical heterogeneity and lack of standardization across the platform utilized, the variability in patient cohorts, and inconsistency in biomarker identification.

A significant portion of the derived evidence stems from retrospective analyses of samples collected during clinical trials, such as the GMMG-MM5 (22), IFM-2009 (30), and Myeloma XI (29) trials. While these studies provide robust longitudinal data, they lack the prospective validation required to prove that altering a treatment plan based solely on MS results improves patient outcomes. Furthermore, the follow-up duration was variable, some reaching medians of over 5 years, and others reporting short follow-up times or not explicitly stating the duration. This may have limited the maturity of survival data, particularly Overall Survival (OS).

As previously alluded to, there is a substantial methodological diversity across the studies, with techniques ranging from MALDI-TOF platforms like EXENT (26, 31) to more complex LC–MS/MS systems like EasyM (25, 28). These platforms demonstrate various levels of analytical sensitivity. For instance, EasyM reported sensitivity up to 1,000-fold higher than SPEP (28), whereas other platforms may have different thresholds. This lack of a universal standardized protocol makes it difficult to compare exact “positive” or “negative” cut-offs between different centers and studies.

The study populations showed diversity, from small discovery cohorts of 26 to 41 patients to larger validation cohorts of over 500 patients. Small sample sizes may limit the generalizability of the metabolomic or proteomic biomarkers to the broader multiple myeloma (MM) population. Not to mention the heterogeneity with regards to the specific MM diagnosis, whether it being newly diagnosed (NDMM), relapsed/refractory (RRMM), or high-risk smoldering (HRsMM) patients, all which may respond differently to MS-based monitoring.

In the prognostic biomarker discovery group, although a consistent theme of dysregulated pathways emerged, such as cellular metabolism (19) and tumor microenvironment (16), no single protein biomarker was consistently identified across all 11 studies (Table 1). This not only highlights the biological complexity of MM, but also suggests that a panel of markers rather than a single protein may be necessary for future risk stratification.

Despite these limitations, the high degree of concordance between blood-based MS and traditional bone marrow MRD, alongside the independent prognostic value of MS-derived biomarkers across various R-ISS stages, underscores the transformative potential of this technology.

### 4.8 Future directions and policy implications

The evidence synthesized here supports several priorities for research, clinical practice, and policy.

Technical Harmonization: International consensus on sensitivity thresholds, quality control metrics, and reporting standards is needed before MS can become standard-of-care. The International Myeloma Working Group (IMWG) should consider establishing a task force to develop guidelines for MS method validation, positivity definitions, and quality assurance, similar to existing guidelines for NGS-MRD.

Interventional Trials: Prospective studies randomizing patients to MS-guided versus conventional monitoring are required to determine whether early intervention upon MS-detected relapse improves outcomes. Such trials should stratify by risk group, as the optimal management strategy may differ between high-risk and standard-risk patients. The lead time of 2–11 months identified by Liyasova et al. (13) provides a window for intervention that should be tested in randomized designs.

Response Criteria Evolution: The cumulative evidence (Tables 1–3) supports consideration by the IMWG of whether MS-based assessment should be incorporated into future iterations of response criteria. Given its demonstrated ability to risk-stratify patients currently classified as IFE-negative complete responders (14, 15), MS could define a new category of “MS-defined deep response” or “blood-based MRD negativity” analogous to bone marrow MRD negativity.

Proteomic Risk Panels: Multi-center validation of a consistent “proteomic risk panel” could enable biology-driven refinement of R-ISS staging. The consistent identification of pathways related to coagulation (34), metabolism (35–38), and microenvironment interaction (39, 40) suggests that a panel approach might outperform single biomarkers. Such a panel could be developed using targeted MS methods and validated in large, diverse cohorts.

Health Economic Analysis: The shift toward MS-based monitoring offers an opportunity to reduce the frequency of invasive BM biopsies. Policy implications include updated monitoring schedules where frequent, less-invasive peripheral blood MS testing is used to “triage” patients for confirmatory bone marrow assessments only when MS signals suggest rising disease. This approach could improve patient quality of life while potentially offering healthcare cost savings by optimizing use of expensive marrow-based sequencing and flow cytometry. Formal health economic analyses are needed to quantify these potential benefits.

Integration with Other Omics: Future research should explore integration of MS proteomics/metabolomics with genomics, transcriptomics, and imaging to develop multi-modal prognostic models. The independent prognostic value of MS biomarkers suggests they capture orthogonal information that could improve existing models.

## 5 Conclusion

In summary, this systematic review of 19 studies demonstrates that quantitative MS-based proteomic and metabolomic profiling provides a high-resolution molecular lens that significantly refines MM risk stratification beyond static staging systems. MS-derived biomarkers have been shown to capture functional disease biology—including metabolic reprogramming, microenvironmental interactions, and treatment resistance mechanisms—that R-ISS cannot assess, with independent prognostic value demonstrated in multivariate models (HR range: 0.25–2.84 across studies). For MRD monitoring, blood-based MS has been shown to achieve 100- to 1,000-fold higher sensitivity than conventional serology, detect molecular relapse 2–11 months earlier than standard methods (median: 4.5 months), and provide prognostic information complementary to bone marrow NGS/NGF, with dual negativity associated with 5-year PFS of 89%. These findings support a paradigm shift from reactive management based on intermittent snapshots to proactive, biology-guided surveillance using minimally invasive liquid biopsies. Realizing this potential as standard-of-care will require international harmonization of MS methodologies and prospective interventional trials validating MS-guided therapeutic decision-making. The evidence synthesized here provides the foundation for these next steps toward integrating MS-based assessment into routine clinical practice for patients with multiple myeloma.

## Data Availability

The original contributions presented in the study are included in the article/Supplementary material, further inquiries can be directed to the corresponding author.
