# Supplementary material for: Mass spectrometry-based proteomics and metabolomics in multiple myeloma: a systematic review of prognostic biomarkers and minimal residual disease monitoring
Source: Front Med (Lausanne). 2026 Apr 22;13:1791030. doi: 10.3389/fmed.2026.1791030 (PMC13143611; doi:10.3389/fmed.2026.1791030)
Supplement: Supplementary file 1 [file Supplementary_file_1.docx]

**Complete Search Strings**

***PubMed Search Terms***

("Multiple Myeloma" [MeSH] OR "Multiple Myeloma" OR "Myeloma" OR "Plasma Cell Myeloma" OR "Plasma Cell Neoplasms")

AND

("Proteomics" [MeSH] OR "Mass Spectrometry" [MeSH] OR "Proteome" OR "Mass Spectr*" OR "LC-MS" OR "MALDI" OR "SRM" OR "MRM" OR "SWATH" OR "Quantitative Proteomics")

AND

("Prognosis" [MeSH] OR "Prognostic" OR "Prognostication" OR "Survival" OR "Overall Survival" OR "Progression-Free Survival" OR "Predictive Value" OR "Biomarker" OR "Predictor")

***Embase Search Terms***

('multiple myeloma'/exp OR 'multiple myeloma') AND ('proteomics'/exp OR 'proteomics') AND ('prognosis'/exp OR 'prognosis' OR 'survival')

***Web of Science Search Terms***

(*"Multiple Myeloma" OR Myeloma OR "Plasma Cell Myeloma" OR "Plasma Cell Neoplasm*")

AND

(*Proteomic* OR "Mass Spectr*" OR "Protein Profiling" OR "LC-MS" OR MALDI OR SRM OR MRM OR SWATH OR "Quantitative Proteomics")

AND

(Prognos* OR Survival OR "Overall Survival" OR "Progression-Free Survival" OR "Predictive Value" OR Biomarker* OR Predictor*)

Filters applied: English language, human studies, original research

Date restrictions: November 2025 cutoff
